# Kinematics of the Normal Knee during Dynamic Activities: A Synthesis of Data from Intracortical Pins and Biplane Imaging

**DOI:** 10.1155/2017/1908618

**Published:** 2017-04-11

**Authors:** Xavier Gasparutto, Florent Moissenet, Yoann Lafon, Laurence Chèze, Raphaël Dumas

**Affiliations:** ^1^University Lyon, Université Claude Bernard Lyon 1, IFSTTAR, LBMC UMR_T9406, 69622 Lyon, France; ^2^Centre National de Rééducation Fonctionnelle et de Réadaptation-Rehazenter, Laboratoire d'Analyse du Mouvement et de la Posture (LAMP), Luxembourg, Luxembourg

## Abstract

Few studies have provided in vivo tibiofemoral kinematics of the normal knee during dynamic weight-bearing activities. Indeed, gold standard measurement methods (i.e., intracortical pins and biplane imaging) raise ethical and experimental issues. Moreover, the conventions used for the processing of the kinematics show large inconsistencies. This study aims at synthesising the tibiofemoral kinematics measured with gold standard measurement methods. Published kinematic data were transformed in the standard recommended by the International Society of Biomechanics (ISB), and a clustering method was applied to investigate whether the couplings between the degrees of freedom (DoFs) are consistent among the different activities and measurement methods. The synthesised couplings between the DoFs during knee flexion (from 4° of extension to −61° of flexion) included abduction (up to −10°); internal rotation (up to 15°); and medial (up to 10 mm), anterior (up to 25 mm), and proximal (up to 28 mm) displacements. These synthesised couplings appeared mainly partitioned into two clusters that featured all the dynamic weight-bearing activities and all the measurement methods. Thus, the effect of the dynamic activities on the couplings between the tibiofemoral DoFs appeared to be limited. The synthesised data might be used as a reference of normal in vivo knee kinematics for prosthetic and orthotic design and for knee biomechanical model development and validation.

## 1. Introduction

Due to ethical and experimental issues, very few studies have provided the in vivo tibiofemoral kinematics of the normal knee. Indeed, the only methods that can accurately provide such information are intracortical pins coupled with the Roentgen stereophotogrammetric analysis of the bones (e.g., [1]), biplane fluoroscopy coupled with computed tomography and three-dimensional (3D) reconstruction of the bones (e.g., [2]), and high-speed stereoradiography with bone-implanted radio-opaque markers (e.g., [3]). These methods are considered the gold standard but are invasive and/or ionising.

The present study aimed at getting a better understanding of the healthy knee in vivo tibiofemoral kinematics during dynamic activities. Indeed, knowledge of the normal in vivo tibiofemoral kinematics is essential to evaluate pathological conditions and surgical treatments or to design knee prosthesis and orthosis that are consistent with a healthy knee. A synthesis of data from intracortical pins and biplane imaging was proposed. Rather than a systematic review, which would have only revealed the inconsistency of the reported data, a reprocessing of the 6 degrees of freedom (DoFs) of the tibiofemoral joint using a standardised method was performed based on mean curves displayed in the published papers. Indeed, various conventions have been used in literature to report the kinematic data of the knee, resulting in contradictory observations and inability to compare data, even if conformation to the general method of Grood and Suntay [4] was most commonly claimed.

Thus, to allow the comparison between datasets with different conventions, the kinematics displayed in studies that used intracortical pins and biplane imaging were transformed into the standardised convention proposed by the International Society of Biomechanics (ISB) [5]. After transformation, as flexion-extension is the main DoF at the knee, the DoFs of the tibiofemoral joint were plotted against the flexion angle such that the patterns of the DoFs during knee flexion could be compared during various dynamic activities, namely, walking, drop landing, hopping, stair ascending, running, and cutting. Then, a clustering method was applied to investigate if the couplings between the DoFs were consistent among the different activities and the measurement methods. The influence of the transformation of the kinematic data into the ISB standards on the couplings between the DoFs was evaluated by computing the determination coefficient (*R*^2^) and the root mean square deviation (RMSD) between the original and transformed data.

## 2. Material and Methods

**Figure 1:**
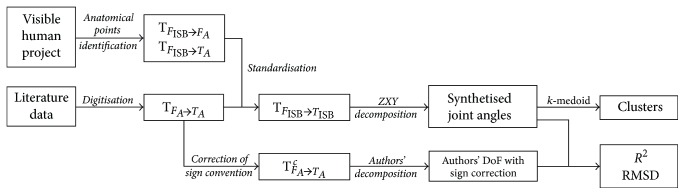
Workflow of the kinematic data processing.

The workflow of the data processing described in the following sections is pictured in Figure 1.

### 2.1. Original Kinematic Data

This study was not a systematic review or a meta-analysis and did not conform to the Prisma guidelines [6]. Nevertheless, the relevant papers were collected using database queries and a citation snowballing procedure (keywords are provided in Appendix). These studies displayed the normal tibiofemoral joint kinematics measured using gold standard methods during weight-bearing dynamic activities: walking [1, 2, 7–10], drop landing [11], hopping [12, 13], stair ascending [14, 15], running [3, 15–17], and cutting [7, 18].

The mean curves of the 6 DoFs of the tibiofemoral joint displayed by the authors were extracted from each paper using an open source digitising software (Engauge Digitiser 4.1, Free Software Foundation). These mean curves presented discrepancy in terms of number of subjects (1 to 30) and gender (male and female). The proximal-distal displacement was found negligible in some studies and thus not reported [9–11, 13, 17]. The characteristics of the author's data are reported in Table 1. All data were expressed in percentage of the relevant movement.

From the digitised joint angles and displacements, the transformation matrices **T**_*F*_*A*_→*T*_*A*__, representing the movement of the tibia relative to the femur within the authors conventions, were computed. Two main authors' conventions were used among the selected studies: the convention described by Lafortune et al. [1], used by the authors measuring with intracortical pins methods, and the convention described by Tashman and Anderst [19], used by the authors measuring with biplane imaging methods, apart from one study [18] that used inertial axes of the bone computed from 3D reconstruction. These two conventions were also adapted in some studies, particularly for the definition of the origins of the femur and tibia segment coordinate systems (SCS) [2, 10, 11, 13, 14, 18]. These adaptations were taken into account in the transformation into the ISB standards.

### 2.2. Transformation into the ISB Standards

**Figure 2:**
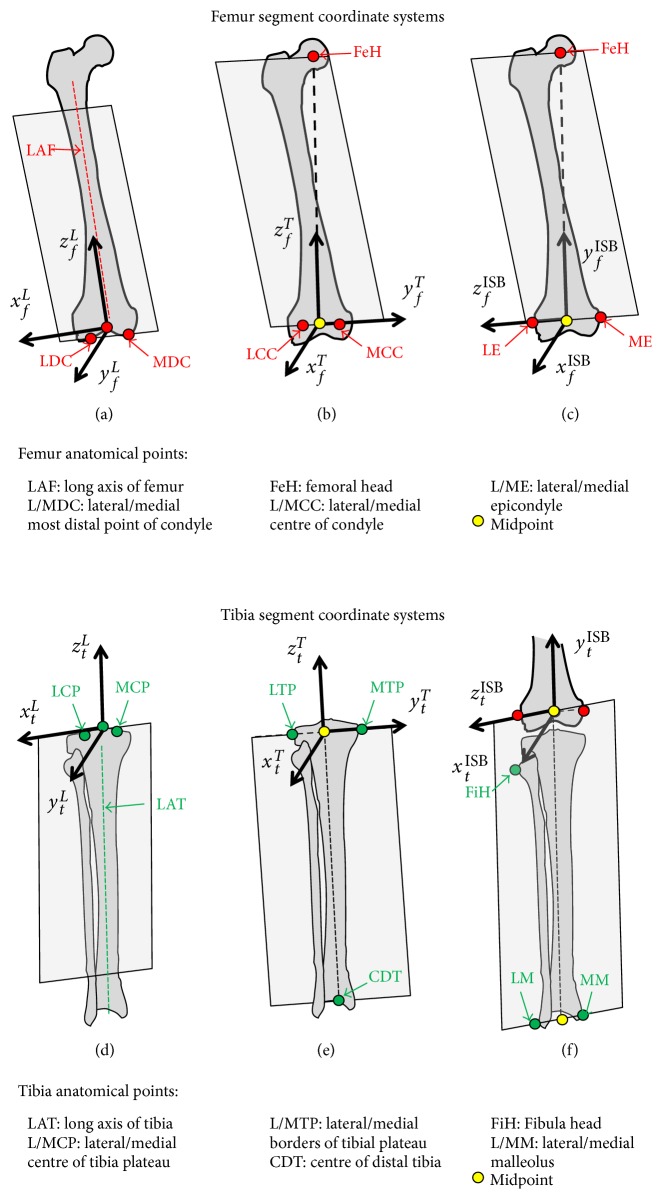
Positions and orientations of the authors' femur and tibia segment coordinate systems (SCS) with respect to the standardised SCS based on the geometry of the Visible Human Project's (VHP) knee: ((a), (d)) Lafortune's convention, ((b), (e)) Tashman's convention, and ((c), (f)) International Society of Biomechanics standards.

To transform the original tibiofemoral kinematics into the ISB standards [5], the positions and orientations of the authors' femur and tibia SCS with respect to the standardised SCS had to be defined (Figure 2). As the knee geometries of the subjects were not available, a reference geometry was used. The right lower limb of the Visible Human Project (VHP) [20] was selected as the extended data are free of charge and were used as reference geometry in numerous studies [21, 22]. The anatomical points used to obtain the SCS of both authors' and standardised conventions were identified on the VHP subject geometry. Based on these points, the transformation matrices between the authors' convention and the ISB standards for the femur (**T**_*F*_ISB_→*F*_*A*__) and tibia (**T**_*T*_ISB_→*T*_*A*__) were defined. Finally, as each subject had different knee anatomy and thus different pose at 0° of flexion, every other DoF was set to zero at 0° of flexion. This was done by superimposing the standardised SCS of the tibia on the standardised SCS of the femur at 0° of flexion [23, 24], with the origin of superimposed femur and tibia SCS in ISB standards defined as the midpoint between femoral epicondyles. The superimposition allowed us to focus on the couplings between the DoFs and not on the absolute values that would have mainly shown the difference in bone orientation at zero degree of flexion. These three steps led to the following transformation matrix:

(1) $$\begin{array}{c} T_{F_{ISB}\to T_{ISB}}=\overset{VHP\;geometry}{\overbrace{T_{F_{ISB}\to F_{A}}}}.\underset{Original\;data}{\underbrace{T_{F_{A}\to T_{A}}}}.\overset{VHP\;geometry}{\overbrace{T_{T_{A}\to T_{ISB}}}}.\underset{\begin{array}{l} Superimposition\\ \;at\;0^{{}^{\circ}}\;of\;flexion \end{array}}{\underbrace{T_{T_{ISB}\to F_{ISB}}^{0}}} \end{array}$$

The 6 DoFs of the tibiofemoral joint were computed from the final transformation matrices **T**_*F*_ISB_→*T*_ISB__, which take into account the joint movement in the author's convention, the change in origin position and axis orientation from the authors' convention to the ISB standards, and the superimposition of the tibia and femur at 0° of flexion. The transformed kinematics represents the movement of the tibia with respect to the femur. The knee joint angles were computed using the joint coordinate system (JCS) equivalent to a *ZXY* Cardanic angle sequence [5]. The displacements of the tibia with respect to the femur were computed as the nonorthonormal projection [25] of the vector from the femur origin to the tibia origin on the axes of the JCS. The positive angles include extension, adduction, and internal rotation, and the positive displacements include lateral, anterior, and proximal. As extension-flexion is the main DoF of the knee, the 5 other DoFs were plotted against the flexion angle to present a synthesis of the kinematic data.

### 2.3. Clustering Method

The synthesised kinematic data (i.e., transformed within the ISB standard) were partitioned into six clusters around medoids [26] with similarity among clusters defined using a “cosine” distance (*kmedoids.m* in Matlab R2015a, The Mathworks). A medoid is defined as the observation of the subset that is the closest to the mean observation within the subset. The idea is to minimise the sum of the distances between each observation of the data's subset and the medoid of the subset. The iterative algorithm returns a cluster index for each observation (as well as cluster's medoid localisation and within-cluster point-to-medoid distances which were not analysed specifically). In the present case, the input data is formed of 17 (i.e., studies) times 100 (i.e., percentage of movement) rows and 6 (i.e., DoFs) columns: **X**_1700×6_. The “cosine” distance between two observations (i.e., rows *i* and *j*) is given by

(2) $$\begin{array}{c} d_{ij}=1-\frac{X_{i}X_{j}^{T}}{\sqrt{\left(X_{i}X_{j}^{T}\right)\left(X_{i}X_{j}^{T}\right)}}. \end{array}$$

The number of clusters, that is, 6, was specifically chosen to test the effect of the activity on the 6-Dof kinematics. Indeed, one cluster by activity would eventually be found if this effect was prevailing. Thus, the proportion of walking, drop landing, hopping, stair ascending, running, and cutting in each cluster was computed. The proportion of intracortical pins, biplane fluoroscopy, and high-speed stereoradiography was also computed to test the effect of the measurement method.

### 2.4. Evaluation of the Transformation

The influence of the transformation of the kinematic data into the ISB standards on the DoF patterns was evaluated with the *R*^2^ and RMSD between the kinematic curves obtained with the transformed (i.e., **T**_*F*_ISB_→*T*_ISB__) dataset and the original dataset corrected for sign convention (i.e., **T**_*F*_*A*_→*T*_*A*__^*c*^, see Figure 1) to avoid large deviations due to differences in axis orientation. As an example, the lateral direction is positive in ISB standards and negative in Tashman's convention.

## 3. Results

**Figure 3:**
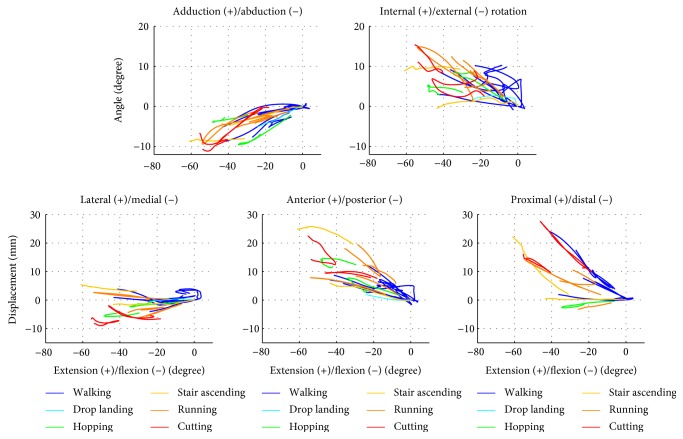
Synthesised tibiofemoral joint angles and displacements during walking, drop landing, hopping, stair ascending, running, and cutting.

The synthesised data are all available in Supplementary Material (synthetised knee kinematic data during weight bearing activities) available online at https://doi.org/10.1155/2017/1908618.  Figure 3 displays the curves of all 17 studies with different colours for each dynamic activity. The range of tibiofemoral extension-flexion was from +4° to −61°. During tibiofemoral flexion, the motion of the tibia relative to the femur was mainly in abduction, internal rotation, and medial, anterior, and proximal displacement. The range of adduction-abduction (AA) was from +1° to −11°. Most of the abduction versus flexion patterns were almost straight lines. The range of the internal-external rotation (IER) was from −1° to +15°. Some internal rotation versus flexion patterns were S-shaped and revealed some differences according to the movement toward flexion or extension. The range of lateral-medial displacement (LM) was from +5 mm to −9 mm. The medial displacement versus flexion patterns were mainly concave curves, with a medial displacement increasing for flexion from approximately 0° to −20° and decreasing for flexion from −20° to −60°. The range of anterior-posterior displacement (AP) was from −2 mm to +26 mm. The anterior displacement versus flexion patterns were mainly convex curves, with an anterior displacement highly increasing for flexion from approximately 0° to −30° and a reduced increase from −30° to −60°. The range of proximal-distal displacement (PD) was from −3 mm to +28 mm. The proximal displacement versus flexion patterns were mainly concave curves, with a proximal displacement slightly increasing for flexion from approximately 0° to −20° and highly increasing from −20° to −60°.

**Figure 4:**
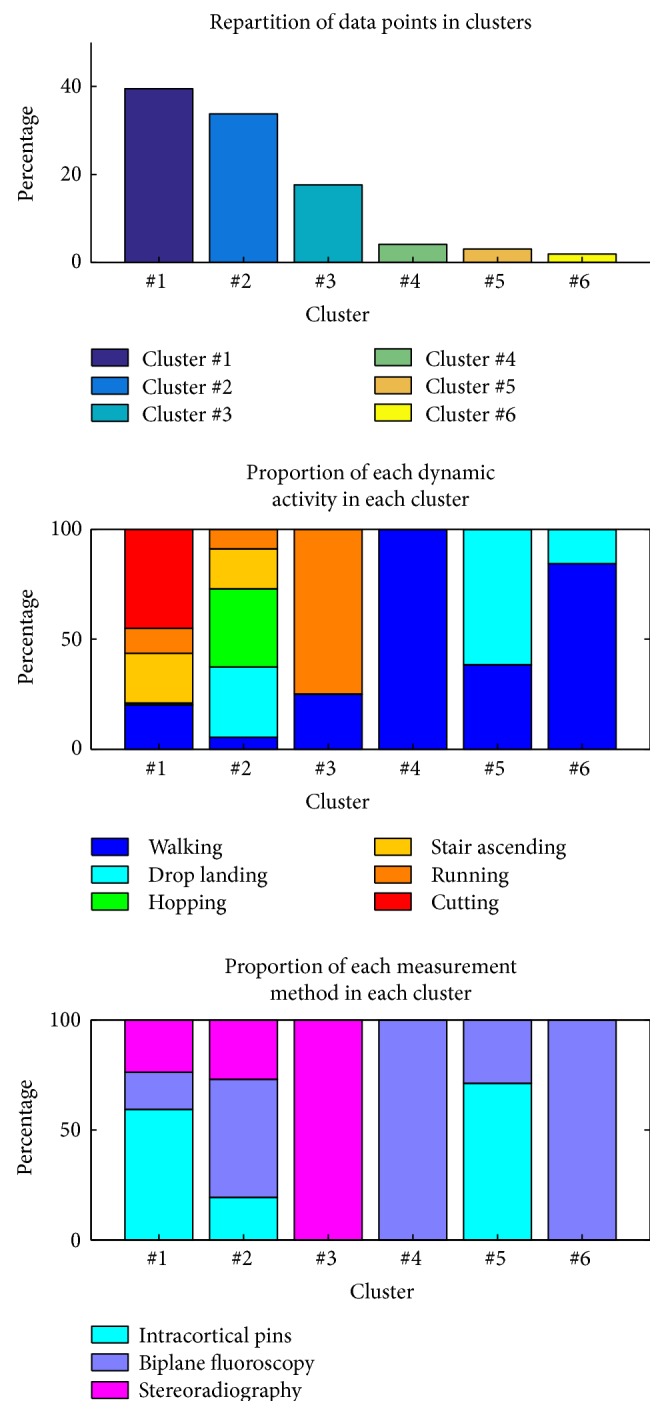
Partition of the synthesised kinematic data into six clusters.

The *k*-medoid procedure resulted in partitioning the data into six clusters (Figure 4) that were not associated to a particular dynamic activity or measurement method except for cluster number 3 (high-speed stereoradiography), number 4 (walking and biplane fluoroscopy), and number 6 (biplane fluoroscopy). The repartition of the whole dataset into the cluster numbers 1 to 6 was 39%, 34%, 18%, 4%, 3%, and 2%, respectively. The curves of all 17 studies with different colours for each cluster are provided as Supplementary Material (Figure S1: repartition of the synthetised knee kinematic data among each cluster).

**Figure 5:**
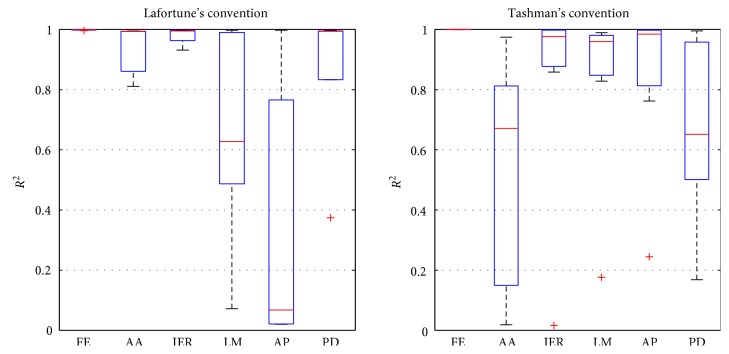
Determination coefficient between the data in the convention of Lafortune and Tashman and the data transformed into the International Society of Biomechanics standards.

Concerning the evaluation of the effect of the transformation of the kinematic data into the ISB standards, the median *R*^2^ (Figure 5) was above 0.9 for the AA, IER, and PD transformed from the studies using Lafortune's convention and for the IER, LM, and AP transformed from the studies using Tashman's convention. It was above 0.6 for the LM transformed from the studies using Lafortune's convention and for the AA and PD transformed from the studies using Tashman's convention. The median *R*^2^ was 0.07 for the AP transformed from the studies using Lafortune's convention.

**Figure 6:**
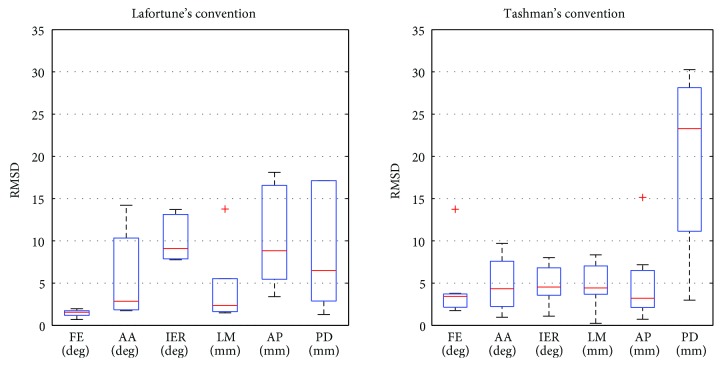
Root mean square difference between the data in the convention of Lafortune and Tashman and the data transformed into the International Society of Biomechanics standards.

The median RMSD (Figure 6) was below 5° for the FE and AA and below 5 mm for the LM transformed from the studies using Lafortune's convention. For the IER, AP, and PD, the median RMSD was below 10° and 10 mm. For the kinematic data transformed from the studies using Tashman's convention, the median RMSD was below 5° and 5 mm except for the PD which reached 23 mm.

## 4. Discussion

In this paper, data from various studies were transformed into the ISB standards [5] to build a homogeneous database of comparable motion and to identify patterns of in vivo tibiofemoral kinematics. The synthesised kinematic data revealed a behaviour that was consistent with the classical functional description of the knee [27–30] which was mainly based on the articular surface and ligament evidences and on in vitro measurements. For instance, the abduction angle (up to −11°) did not demonstrate any decoaptation of the joint, but the difference between the medial and lateral radii of curvature of the femoral condyles and tibial plateaus [29, 31]. Similarly, the proximal displacement (up to 28 mm) did not demonstrate any compression of the femur on the tibia but a variation of the radius of curvature of the femoral condyles and tibial plateaus in the sagittal plane [29, 31]. The literature suggested that normal in vivo kinematics was different from in vitro ones, and varied with dynamic activities, especially for internal-external rotation and anterior-posterior displacement [32–34]. In the present study, three quarters of the synthesised kinematic data were included in two main clusters featuring all dynamic activities. Thus, the effect of the dynamic activities on the couplings between the tibiofemoral DoFs appeared limited. S-shaped patterns in the internal rotation curves were observed in studies using intracortical pins [1, 7, 8] and biplane fluoroscopy [10, 18], but not for studies with high-speed stereoradiography [3, 9, 12, 13, 17]. Moreover, the last quarter of the data was partitioned according to the measurement methods (cluster numbers 3, 4, and 6). Thus, it appeared that the measurement method might yield, in some cases, specific kinematic patterns.

The present study has several limitations. First, the original data correspond to mean curves that do not represent the kinematics of a specific subject [35]. In addition, all the curves were digitised from published graphs. However, this represents currently the only data available in the literature. Second, since they did not exhibit any pathology, the knee joints were assumed normal although some of them were belonging to athletes, seniors, and subjects with contralateral disorders (Table 1). Third, the reprocessing of kinematics (i.e., positions and orientations of the authors' femur and tibia SCS with respect to the standardised SCS) was based on a single cadaveric knee geometry. The bias introduced by this approximation in the geometry cannot be assessed because the geometry of the subjects from the various studies is unknown. However, the variability due to this geometrical approximation should be reduced by the superimposition of the femur and tibia SCS at 0° of flexion [23]. This superimposition is classic when looking at couplings between the DoFs and was previously performed by some authors of the selected studies with superimposition of the origin [9, 11, 13] or without [1, 7, 8]. As an attempt to estimate the variability due to the unknown differences in geometry, the RMSD between the transformed and original data were computed and analysed. The median RMSD was generally below 5° and 10 mm. It is expected that the effect of different geometries is largely inferior to the effect of different segment axes and origin conventions.

As a conclusion, the synthesised kinematic data provide a large sample of couplings between the tibiofemoral DoFs based on 17 studies averaging 126 subjects. The main part of the data was consistent among the different activities (i.e., walking, drop landing, hopping, stair ascending, running, and cutting) and measurement methods (i.e., intracortical pins, biplane fluoroscopy, and high-speed stereoradiography). Moderate to good correlations between the transformed and original data indicated that the patterns of the couplings between the DoFs observed in this study remained generally consistent with the original data. It can be expected that kinematics of normal knee measured with gold standard measurement methods become available as open data in the next future. Meanwhile, the kinematic data synthesised in the present study might be used as a reference of normal in vivo knee kinematics for prosthetic and orthotic design and for the development and validation of biomechanical knee models.

## Supplementary Material

Synthetised knee kinematic data during weight bearing activities.

## Figures and Tables

**Table 1:** Overview of the selected studies: dynamic activities, gold standard measurement methods, subject characteristics, and conventions used for kinematic processing.

Dynamic activity	Author(s)	Year	Gold standard measurement method	Subject characteristics	Convention used for kinematic processing
Walking	Lafortune et al.	1992	Intracortical pins	5 healthy males (27.2 years, 180.6 cm, 75.2 kg)	Lafortune
	Benoit et al.	2006	Intracortical pins	1 healthy male (32 years, 171 cm, 86 kg)	Lafortune
		2007		6 healthy males (26 ± 4.7 years, 176.6 ± 4 cm, 76.3 ± 12.3 kg)	
	Kozanek et al.	2009	Biplane fluoroscopy	6 healthy males, 2 healthy females (32–49 years)	Tashman
	Li et al.	2009	Biplane fluoroscopy	1 healthy male (45 years)	Tashman
	Farrokhi et al.	2012	High-speed stereoradiography with bone-implanted radio-opaque markers	6 males, 6 females (70.2 ± 8 years, 173.3 ± 12.8 cm, 76.7 ± 20.3 kg), healthy (control group)	Tashman

Drop landing	Torry et al.	2011	Biplane fluoroscopy	6 males (34.1 ± 7.9 years, 185 ± 5 cm, 85.1 ± 7.3 kg), recreational athletes	Tashman

Hopping	Beillas et al.	2004	High-speed stereoradiography with bone-implanted radio-opaque markers	1 male (30 years, 172 cm, 75 kg), contralateral knee of unilateral ACL rupture	Tashman
	Deneweth et al.	2010	High-speed stereoradiography with bone-implanted radio-opaque markers	6 males, 3 females (28.8 ± 12.8 years, 174.5 ± 8.9 cm, 77 ± 10 kg), contralateral knee of unilateral ACL rupture, recreational athlete	Tashman

Stair ascending	Kozanek et al.	2011	Biplane fluoroscopy	11 females, 19 males (36 years, 82 kg, 175 cm), contralateral knee of unilateral ACL rupture	Tashman
	Li et al.	2012	High-speed stereoradiography with bone-implanted radio-opaque markers	10 subjects, gender not detailed (21.7 ± 3.6 years, 178.9 ± 8.5 cm, 78.4 ± 17.2 kg), contralateral knee of unilateral grade II PCL injury	Tashman

Running	Reinschmidt	1996	Intracortical pins	3 males (27.7 ± 2.1 years, 1.88 ± 0.10 m, 85.5 ± 9.6 kg), injury free	Lafortune
	Tashman et al.	2004	High-speed stereoradiography with bone-implanted radio-opaque markers	4 females, 2 males (39.6 ± 8.2 years), contralateral knee of ACL reconstruction (4 to 12 months after surgery)	Tashman
		2007		6 females, 10 males (35.4 ± 7.1 years) contralateral knee of ACL reconstruction (1 year after surgery)	
	Li et al.	2012	High-speed stereoradiography with bone-implanted radio-opaque markers	10 subjects, gender not detailed (21.7 ± 3.6 years, 178.9 ± 8.5 cm, 78.4 ± 17.2 kg), contralateral knee of unilateral grade II PCL injury	Tashman

Cutting	Benoit et al.	2006	Intracortical pins	1 healthy male (32 years, 171 cm, 86 kg)	Lafortune
	Miranda et al.	2013	Biplane fluoroscopy	1 typical subject among 5 males and 5 females (25 ± 5.5 years, 1.73 ± 0.10 m, 73.17 ± 10.15 kg), recreational athletes	Other

## Appendix

The keywords used for database queries (Pubmed, Scopus, and Web of Knowledge) were biplan^∗^ fluoroscop^∗^ OR dual fluoroscop^∗^ stereo X-ray OR stereo^∗^radiograph^∗^ OR biplan^∗^ radiograph^∗^ OR pins AND knee OR tibio^∗^femoral AND kinematic^∗^ AND gait OR walking OR running OR stair OR step OR cut^∗^ OR jump OR hop^∗^ OR drop. From the obtained references, the selected papers were those displaying all of the 6 DoFs of normal tibiofemoral joint.
