# Supplementary figures and images for: Kinematics of the Normal Knee during Dynamic Activities: A Synthesis of Data from Intracortical Pins and Biplane Imaging
Source: Appl Bionics Biomech. 2017 Apr 11;2017:1908618. doi: 10.1155/2017/1908618 (PMC5405570; doi:10.1155/2017/1908618)

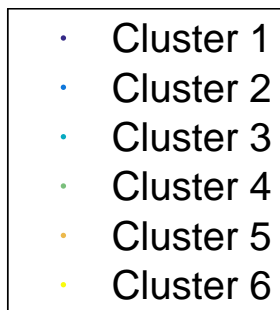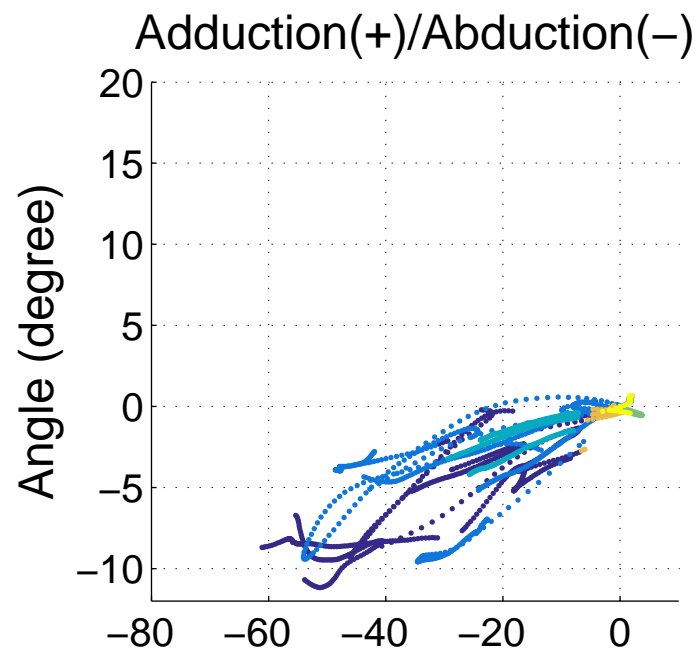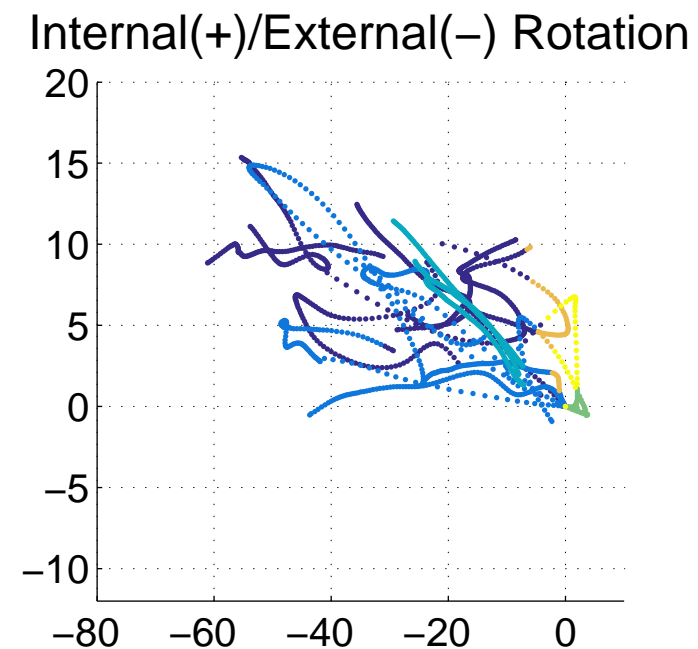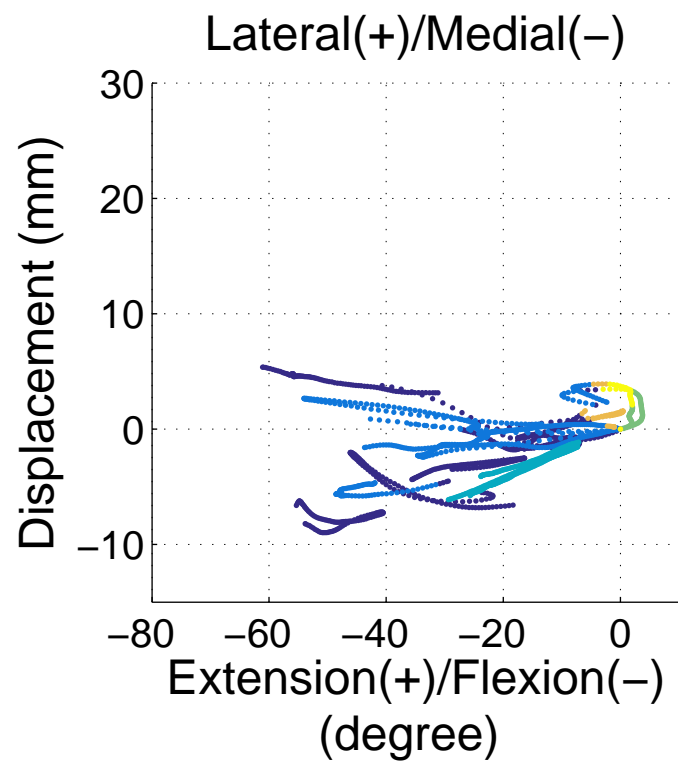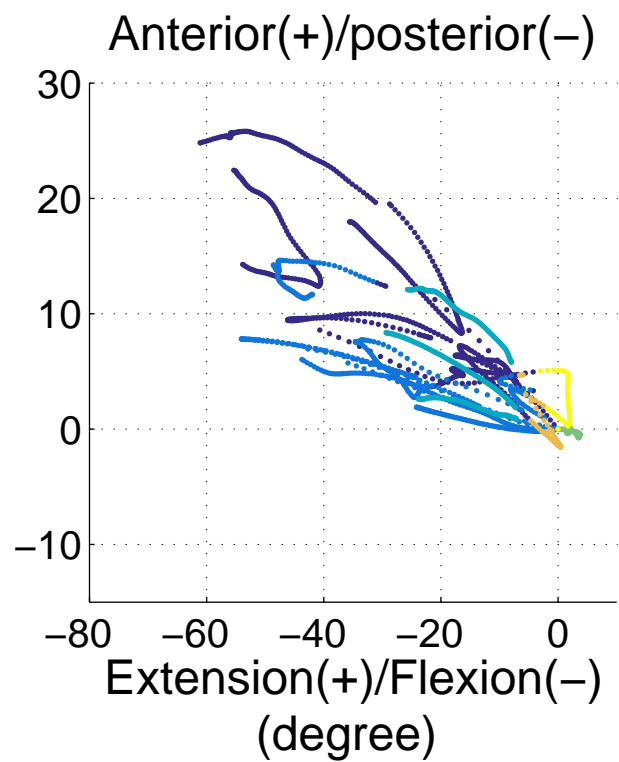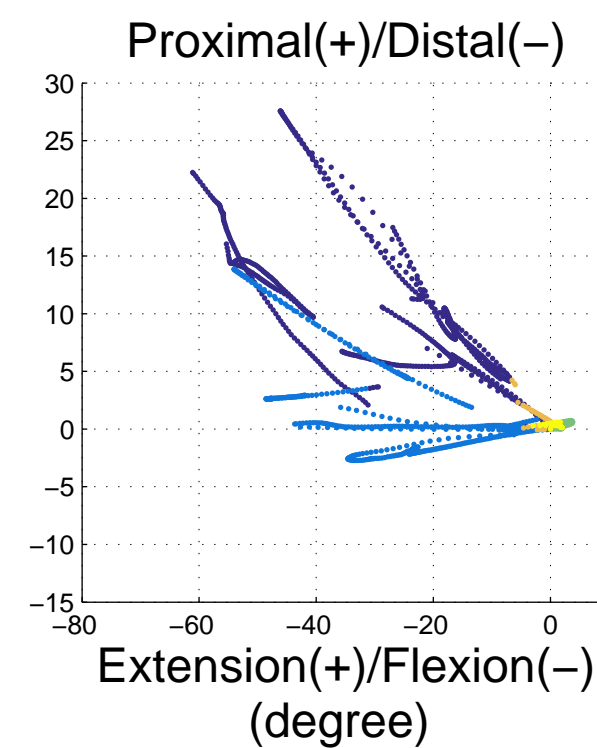

Supplement: Supplementary file 1 — Synthetised knee kinematic data during weight bearing activities. [file 1908618.f1.pdf]
